# Bispecific Antibodies and Antibody–Drug Conjugates in Relapsed/Refractory Aggressive Non-Hodgkin Lymphoma, Focusing on Diffuse Large B-Cell Lymphoma

**DOI:** 10.3390/cancers17152479

**Published:** 2025-07-26

**Authors:** Santino Caserta, Chiara Campo, Gabriella Cancemi, Santo Neri, Fabio Stagno, Donato Mannina, Alessandro Allegra

**Affiliations:** 1 Hematology Unit, Department of Human Pathology in Adulthood and Childhood “Gaetano Barresi”, University of Messina, Via Consolare Valeria, 98125 Messina, Italy; santino.caserta@polime.it (S.C.); fabio.stagno@unime.it (F.S.); 2 Hematology Unit, Oncology-Hematology Department, Azienda Ospedaliera Papardo, 98158 Messina, Italy; chiaracampo@aopapardo.it (C.C.); gabriellacancemi@aopapardo.it (G.C.); santoneri@aopapardo.it (S.N.);

**Keywords:** bispecific antibodies, epcoritamab, glofitamab, antibody–drug conjugates, polatuzumab vedotin, loncastuximab tesirine, non-Hodgkin lymphoma, relapsed/refractory non-Hodgkin lymphoma, aggressive lymphomas, immunotherapy

## Abstract

Relapsed/refractory diffuse large B-cell lymphoma and other non-Hodgkin lymphomas represent significant clinical challenges, particularly in patients who have exhausted standard immunochemotherapy and cellular therapies. Bispecific antibodies and antibody–drug conjugates have emerged as promising treatments, offering targeted and more effective treatment options compared to current standards. Bispecific antibodies, including epcoritamab and glofitamab, third-line therapies for diffuse large B-cell lymphoma, are recombinant immunoglobulins engineered to recognize two distinct antigens or epitopes simultaneously. This capability enhances therapeutic precision by bridging immune effector cells and tumor cells and modulating multiple signaling pathways involved in the pathogenesis of non-Hodgkin lymphoma. In the context of new therapies, antibody–drug conjugates, such as loncastuximab tesirine, are therapies composed of monoclonal antibodies linked to cytotoxic agents, in which the antibody selectively binds to tumor-associated antigens, delivering the cytotoxic payload directly to cancer cells while minimizing off-target effects. They combine the specificity of antibodies with the potency of chemotherapy, offering enhanced efficacy and safety in hematological malignancies. Ongoing clinical trials are investigating other molecules like odronextamab and the use of bispecific antibodies in combination regimens and earlier lines of therapy. The aim of this review is to explore actual therapies in relapsed/refractory diffuse large B-cell lymphoma, focusing on bispecific antibodies and antibody–drug conjugates.

## Simple Summary

Relapsed or refractory diffuse large B-cell lymphoma and other non-Hodgkin lymphomas present major therapeutic challenges, especially in patients who have exhausted standard immunochemotherapy and cellular therapies. Recent advances in immuno-oncology have introduced bispecific antibodies and antibody–drug conjugates as innovative treatment strategies. Bispecific antibodies, such as epcoritamab and glofitamab, engage both tumor-associated antigens and immune effector cells, enhancing cytotoxicity and clinical efficacy in heavily pretreated diffuse large B-cell lymphoma. Antibody–drug conjugates, including loncastuximab tesirine, deliver cytotoxic agents directly to malignant cells through antigen-specific monoclonal antibodies, improving tumor selectivity and minimizing off-target effects. Ongoing clinical trials are investigating additional molecules, evaluating their integration into earlier treatment lines and combination regimens. This review aims to provide an overview of current and emerging therapeutic approaches in relapsed/refractory diffuse large B-cell lymphoma, with a focus on bispecific antibodies and antibody–drug conjugates.

## 1. General Considerations Regarding Bispecific Antibodies and Antibody–Drug Conjugates

### 1.1. Bispecific Antibodies

The concept of bispecific antibodies (BsAbs) was first introduced in the 1960s, when Nisonoff and colleagues hypothesized the possibility of creating antibody molecules capable of simultaneously binding two distinct antigens [1]. However, the clinical translation of this idea required decades of technological progress.

Early bispecific constructs, created in the 1980s and 1990s, had significant limitations related to molecular stability, production scalability, and immunogenicity [2]. The advent of recombinant DNA engineering and humanized antibody platforms led to the development of new BsAb formats with improved pharmacokinetics, reduced immunogenicity, and greater manufacturing efficiency [3,4]. Several innovative platforms emerged, including bispecific T-cell engagers (BiTEs), dual-affinity re-targeting (DART), tandem diabodies (TandAbs), and IgG-like bispecific antibodies with engineered Fc regions [5].

The clinical potential of BsAbs was first realized in B-cell malignancies, where the stable and homogeneous expression of surface antigens such as CD19 and CD20 provided ideal therapeutic targets [6]. This progress culminated in the development of blinatumomab, the first BsAb to receive regulatory approval and provide clinical proof of concept for the T-cell redirection strategy [7].

Blinatumomab, a BiTE targeting CD19 and CD3, was approved by the Food and Drug Administration (FDA) in 2014 for relapsed/refractory (R/R) B-precursor acute lymphoblastic leukemia (ALL) [7]. Its clinical success—demonstrating the ability to achieve deep molecular remissions and minimal residual disease (MRD) negativity—validated the BsAb approach and paved the way for the development of second- and third-generation BsAbs specifically optimized for the treatment of B-cell non-Hodgkin lymphomas (B-NHL) [8,9,10,11].

**Figure 1:**
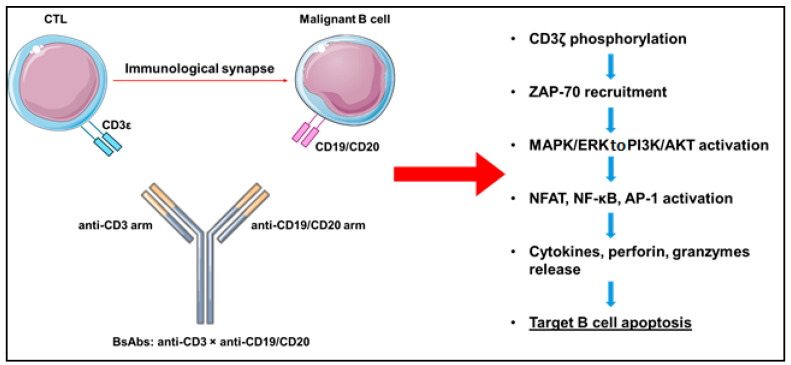
Mechanism of action of BsAbs. CTL, cytotoxic T lymphocytes; BsAbs, bispecific antibodies. This figure was partly generated using Servier Medical Art, provided by Servier, licensed under a Creative Commons Attribution 3.0 Unported License.

Mechanistically, BsAbs act by forming an artificial immunological synapse between cytotoxic T lymphocytes (CTLs) and malignant B cells. The anti-CD3 arm of the BsAb binds to the ε subunit of the T-cell receptor (TCR) complex on T lymphocytes, while the anti-CD19 or anti-CD20 arm binds to the corresponding antigen on the surface of B cells [12,13] (Figure 1). This interaction is independent of major histocompatibility complex (MHC) class I, overcoming a common immune escape mechanism in lymphomas, where tumor cells downregulate MHC expression [14,15].

The establishment of the immunological synapse initiates T-cell activation via a series of intracellular signaling events, beginning with the phosphorylation of the CD3ζ chain and recruitment of the kinase ZAP-70. This triggers downstream signaling through the MAPK/ERK and PI3K/AKT pathways, ultimately leading to calcium influx and the activation of transcription factors such as NFAT, NF-κB, and AP-1, which collectively drive T-cell proliferation, cytokine secretion, and functional activation. Activated T cells then release cytotoxic granules containing perforin and granzymes—especially granzyme B—which induce apoptosis in target B cells through the mitochondrial (intrinsic) apoptotic pathway [3,16]. Concurrently, pro-inflammatory cytokines, including IFN-γ, TNF-α, and IL-2, are secreted, enhancing the anti-tumor response and facilitating the recruitment of additional immune cells into the tumor microenvironment [2,17]. The efficacy and modulation of this immune response are highly dependent on the molecular architecture of BsAbs. Engineering modifications to the Fc domain—specifically to abrogate Fcγ receptor binding—minimize off-target systemic immune activation, and fine-tuning CD3-binding affinity helps optimize T-cell engagement while reducing the risk of excessive cytokine release [10,11].

Glofitamab, for instance, utilizes a 2:1 design—bivalent for CD20 and monovalent for CD3—yielding enhanced avidity, improved immunological synapse formation, and sustained cytotoxic activity. In contrast, BsAbs such as epcoritamab and mosunetuzumab adopt 1:1 or tandem single-chain variable fragment (scFv) configurations, which confer structural flexibility and superior tissue penetration, particularly advantageous in patients with large nodal disease burdens [18,19].

Clinically, the landscape of BsAb therapy for B-NHL has evolved rapidly. Glofitamab, evaluated in the NP30179 trial, demonstrated an overall response rate (ORR) of 56% and a complete response (CR) rate of 43% among patients with R/R diffuse large B-cell lymphoma (DLBCL), including those previously treated with chimeric antigen receptor T cells (CAR-Ts) [3].

Epcoritamab, administered subcutaneously, showed an ORR of 63.1% and a CR of 40.1% in the EPCORE NHL-1 study, with highly favorable 2-year survival rates. In detail, utilizing data from two Phase I/II trials (EPCORE^®^ NHL 1 and NHL 3), the authors developed a robust PK model characterizing absorption, distribution, and clearance: the analysis identified key covariates influencing systemic exposure and supported dose optimization to maximize therapeutic index while minimizing toxicity. These findings underpin dosing strategies for epcoritamab in this challenging patient population [12,19].

The clinical success of these BsAbs highlights their role as a new class of “off-the-shelf” immunotherapies, offering immediate availability, repeat dosing, and outpatient administration [18]. This feature is particularly attractive for patients ineligible for CAR-T therapy or those with early relapse after CAR-T [20].

Despite these successes, several challenges remain. CRS, a systemic inflammatory response caused by the rapid activation and proliferation of immune effector cells, leads to a massive release of pro-inflammatory cytokines. Clinically, it presents with a wide range of symptoms, from mild fever, fatigue, and myalgias to severe manifestations such as hypotension, hypoxia, coagulopathy, and multi-organ dysfunction. It is a common and potentially life-threatening complication of T cell-engaging therapies, including bispecific antibodies and CAR-T cells, and requires prompt recognition and management. Standard treatment includes supportive care, corticosteroids, and cytokine-targeting agents such as tocilizumab, particularly in moderate to severe cases [18]. Immune effector cell-associated neurotoxicity syndrome (ICANS) is a serious neurological complication that can become life-threatening when observed with bispecific antibodies that recruit T cells, such as CD3 × CD20 or CD3 × BCMA constructs. ICANS is characterized by a range of clinical symptoms, including headache, confusion, aphasia, seizures, and in severe cases, cerebral edema and coma. The exact pathophysiology is not fully understood, but it is believed to involve excessive cytokine release, endothelial activation, and disruption of the blood–brain barrier, leading to neuroinflammation. Risk factors for ICANS include high tumor burden, rapid T-cell activation, and concomitant CRS. The frequency and clinical severity of ICANS are influenced by the structural format of the bispecific antibody, its administration route, and the dosing regimen employed. Standard management strategies include corticosteroid therapy, seizure prophylaxis, and close neurological monitoring. Early recognition and timely intervention are essential to mitigate the risk of irreversible neurotoxicity. As the use of bispecific antibodies continues to expand across hematologic malignancies, a deeper understanding of ICANS pathophysiology and risk factors will be crucial to optimizing patient safety and therapeutic outcomes [13,15]. The introduction of step-up dosing protocols and premedications has improved safety [14], but careful clinical management remains essential, particularly in frail patients [12].

Another critical challenge is the loss or downregulation of the target antigen (particularly CD20) [15], which can emerge as a clonal resistance mechanism during BsAb therapy [13]. In fact, this condition represents a key clonal resistance mechanism during bispecific antibody therapy, which relies on the stable and sufficient expression of CD20 on malignant B cells to facilitate effective immune synapse formation and T cell-mediated cytotoxicity. However, selective pressure exerted by BsAb treatment can lead to immune editing, promoting the survival and expansion of tumor subclones with reduced or absent CD20 expression. This antigen escape impairs BsAb binding and limits T-cell engagement, ultimately resulting in disease relapse or progression. Mechanisms underlying CD20 downregulation include epigenetic modifications, transcriptional repression, and internalization or shedding of the antigen. In some cases, prior exposure to anti-CD20 monoclonal antibodies may also contribute to CD20 modulation. Monitoring antigen expression over time and combining BsAbs targeting multiple antigens may help prevent or overcome this resistance, improving the durability of clinical responses. Innovative strategies include the use of multispecific BsAbs (e.g., anti-CD19/CD20), optimal sequencing of treatments, and combination with checkpoint inhibitors [18].

**Figure 2:**
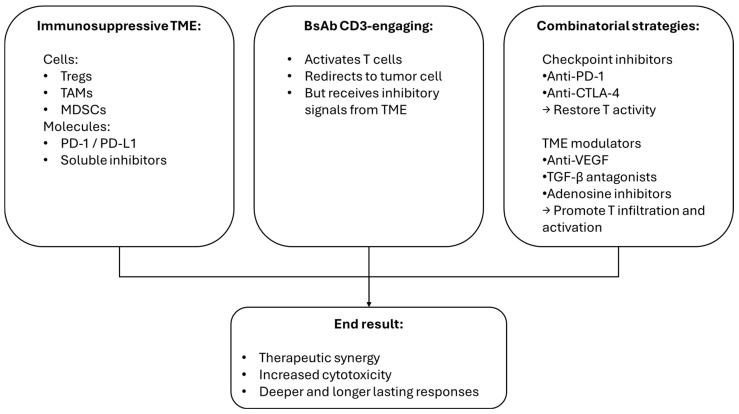
Strategies to overcome immunosuppressive barriers in the TME and enhance the activity of bispecific antibodies.

Finally, the immunosuppressive tumor microenvironment (TME) represents a major barrier [2]. The presence of regulatory T cells (Tregs), tumor-associated macrophages (TAMs), and immune checkpoints such as PD-1/PD-L1 limits BsAb efficacy. Combining BsAbs with immune-checkpoint inhibitors or agents that modulate the TME represents a promising strategy to enhance anti-tumor efficacy and overcome resistance. BsAbs, such as CD3-engaging constructs, activate T cells and redirect them toward tumor cells, but their function can be limited by immunosuppressive signals within the TME, including PD-1/PD-L1 interactions and the presence of regulatory T cells, myeloid-derived suppressor cells, and inhibitory cytokines. Co-administration of checkpoint inhibitors (e.g., anti-PD-1 and anti-CTLA-4) can restore T-cell activity and prevent exhaustion, thereby amplifying BsAb-induced cytotoxicity. Additionally, agents that remodel the TME—such as inhibitors of adenosine signaling, VEGF blockers, or TGF-β antagonists—can further support immune infiltration and activation. Preclinical studies and early-phase clinical trials demonstrate synergistic effects with combination approaches, leading to deeper and more durable responses. Rational design of combination regimens, with attention to toxicity and timing, may significantly expand the therapeutic potential of BsAbs in solid and hematologic tumors [10,18] (Figure 2).

In conclusion, the clinical trajectory of BsAbs is rapidly evolving. Following the pioneering success of blinatumomab, next-generation BsAbs such as glofitamab and epcoritamab are already transforming the treatment of B-cell lymphomas. The coming years will see their integration into combination regimens and earlier lines of therapy, with the goal of maximizing the depth and durability of responses and improving overall survival for patients.

BsAbs and ADCs represent two complementary strategies in the treatment landscape of relapsed/refractory non-Hodgkin lymphomas, and their integration or sequential use may enhance efficacy and delay resistance.

### 1.2. Antibody–Drug Conjugates

Antibody–drug conjugates (ADCs) represent a sophisticated class of targeted cancer therapeutics that has garnered significant attention for the treatment of hematologic malignancies, particularly B-cell lymphomas.

The conceptual framework for ADCs dates to the late 1970s, when researchers first envisioned utilizing monoclonal antibodies as delivery vehicles for potent cytotoxic agents [21]. However, technical hurdles—including instability of early antibody constructs, lack of sufficiently potent and selective cytotoxic payloads, and suboptimal linker chemistry—impeded progress for decades [22]. The clinical landscape changed dramatically with the regulatory approval of gemtuzumab ozogamicin (GO) in 2000 for CD33-positive acute myeloid leukemia (AML) [23]. Although GO was temporarily withdrawn due to hepatotoxicity, subsequent optimization of its dosing strategy and further refinement of ADC technologies laid the groundwork for the modern generation of ADCs [24].

**Figure 3:**
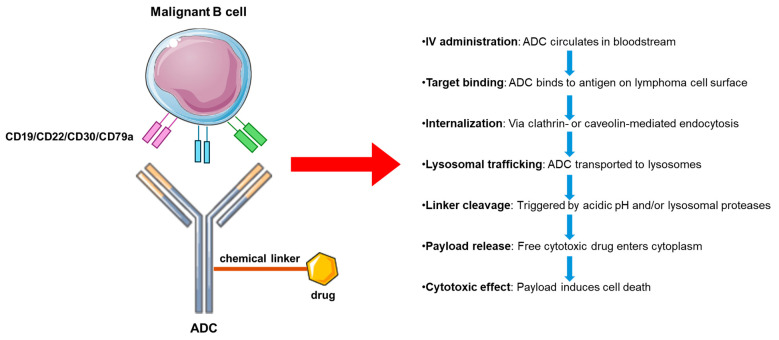
Mechanism of action of ADCs. ADC, antibody–drug conjugate. This figure was partly generated using Servier Medical Art, provided by Servier, licensed under a Creative Commons Attribution 3.0 Unported License.

ADCs are designed to maximize tumor-specific cytotoxicity while minimizing off-target toxicity [25]. This is achieved through an intricate three-part architecture: a monoclonal antibody that selectively targets tumor-associated antigens, a chemical linker that maintains conjugate stability in circulation but facilitates payload release within tumor cells, and a highly potent cytotoxic agent. In the context of B-cell lymphomas, optimal antigen targets include proteins with high, homogeneous expression on malignant B cells and limited expression on normal tissues—such as CD30, CD79b, CD19, and CD22 [26,27,28] (Figure 3).

Following intravenous administration, the ADC circulates until it binds its cognate antigen on the surface of lymphoma cells [29]. The antibody–antigen complex is then internalized via clathrin-mediated or caveolin-mediated endocytosis [30]. Upon internalization, the ADC traffics to the lysosomal compartment, where the acidic environment and/or lysosomal proteases cleave the linker [31]. This cleavage event releases the free cytotoxic payload into the cytoplasm, where it can exert its lethal effects [32].

The payloads used in ADCs for lymphoma therapy are among the most potent cytotoxins known, often with picomolar IC_50_ values [33]. These include tubulin polymerization inhibitors such as monomethyl auristatin E (MMAE), DNA-alkylating agents such as pyrrolobenzodiazepine (PBD) dimers, and calicheamicin derivatives [34]. MMAE binds to tubulin, disrupting microtubule dynamics, arresting the cell cycle in the G_2_/M phase, and inducing apoptosis through mitotic catastrophe [35]. PBD dimers covalently crosslink the DNA minor groove, preventing the DNA strand separation required for replication and transcription, leading to double-strand breaks and apoptotic cell death [36]. Calicheamicins similarly induce DNA strand breaks through radical-mediated cleavage mechanisms [37].

An important and therapeutically beneficial feature of ADCs is the so-called “bystander effect” [38]. Certain payloads and linker combinations allow diffusion of the active cytotoxic drug into neighboring cells, which may be antigen-negative or express antigen at low density. This phenomenon can overcome the challenge of antigen heterogeneity within the tumor mass—a particular concern in DLBCL, where antigen modulation and clonal evolution contribute to treatment resistance [39].

In addition to direct cytotoxicity, the Fc portion of the antibody component may engage Fcγ receptors on immune effector cells, triggering antibody-dependent cellular cytotoxicity (ADCC) and phagocytosis [40]. This immune engagement further amplifies the anti-tumor effect and may contribute to long-term immunologic control of minimal residual disease.

Clinically, ADCs have transformed the treatment landscape for B-NHL. Brentuximab vedotin (anti-CD30-MMAE) was the first ADC approved for the treatment of lymphoma and remains a cornerstone of therapy for CD30-positive malignancies [41]. Initially approved for R/R classical Hodgkin lymphoma (cHL) and systemic anaplastic large-cell lymphoma (sALCL), brentuximab vedotin is now incorporated into first-line treatments for advanced cHL based on the ECHELON-1 trial, which demonstrated superior progression-free survival compared to ABVD chemotherapy (doxorubicin (Adriamycin), bleomycin, vinblastine, and dacarbazine) in patients with stage III or IV cHL [42]. Notably, brentuximab vedotin has shown activity in CD30-positive NHLs, including primary mediastinal large B-cell lymphoma (PMBCL) and certain subsets of DLBCL [43].

Polatuzumab vedotin (anti-CD79b-MMAE) has significantly improved outcomes in relapsed/refractory diffuse large B-cell lymphoma (R/R DLBCL). In the Phase II GO29365 trial, the Pola-BR regimen (polatuzumab vedotin, bendamustine, and rituximab) achieved a 63% overall response rate and a 40% complete response rate, outperforming BR alone, with durable responses and manageable toxicity. This led to its accelerated approval for R/R DLBCL after at least two prior therapies. Polatuzumab is also being evaluated in frontline regimens like pola-R-CHP, aiming to enhance efficacy and reduce the vincristine-associated neurotoxicity seen in R-CHOP. However, a U.S. survey of 503 oncology professionals revealed systemic barriers to therapy access: 46.6% observed treatment abandonment due to financial toxicity, and 31.5% noted a lack of adherence monitoring. Delays from prior authorizations and administrative burden further complicate treatment delivery. These findings underscore the need for improved access, cost management, and adherence strategies to optimize the impact of novel agents like polatuzumab vedotin [44,45,46,47].

Loncastuximab tesirine (anti-CD19-PBD dimer) is an emerging ADC that expands treatment options for R/R DLBCL, including in patients who have failed CAR-T-cell therapy. The LOTIS-2 trial demonstrated an ORR of 48.3% and a CR rate of 24.1%, with responses observed across high-risk subgroups [43]. The ability of loncastuximab to induce remissions post-CAR-T highlights the potential of ADCs to address refractory disease mediated by T-cell dysfunction or immune escape. In their study, Caimi and colleagues present data from an extended follow-up of patients treated with loncastuximab tesirine, showing durable responses with manageable safety profiles, providing a promising therapeutic option for this challenging patient population. These findings support its potential integration into treatment regimens for relapsed/refractory DLBCL [48].

Inotuzumab ozogamicin (InO) is a targeted therapy for relapsed or refractory B-cell acute lymphoblastic leukemia (B-ALL). It comprises a humanized anti-CD22 monoclonal antibody conjugated to N-acetyl-γ-calicheamicin, a potent cytotoxic agent derived from *Micromonospor aechinospora*. Upon binding to CD22-expressing leukemic cells, InO is internalized, and the cytotoxic agent is released intracellularly. This agent induces double-strand DNA breaks, leading to cell-cycle arrest and apoptosis, independent of cell-cycle phase. Clinically, InO has demonstrated superior efficacy compared to standard chemotherapy regimens, with higher complete remission rates and prolonged progression-free survival. However, its use is associated with risks, including veno-occlusive liver disease, necessitating careful patient selection and monitoring. InO represents a significant advancement in the treatment of B-ALL, offering a more targeted approach with improved outcomes for patients with limited treatment options [49]. Preliminary studies suggest meaningful activity in indolent and aggressive B-cell lymphomas, warranting further exploration [50].

Beyond these approved agents, numerous ADCs targeting novel antigens (e.g., CD37, CD70, CD25) are under active clinical development in B-NHL. Camidanlumab tesirine (Cami) is an antibody–drug conjugate targeting CD25, the alpha chain of the interleukin-2 receptor, expressed on activated T cells. It comprises a human IgG1 monoclonal antibody conjugated to a pyrrolobenzodiazepine dimer cytotoxic agent, SG3199. Upon binding to CD25-expressing cells, Cami is internalized, and SG3199 induces DNA interstrand crosslinks, leading to cell death. In a pivotal Phase II trial involving 117 patients with relapsed or refractory classical Hodgkin lymphoma (cHL), Cami demonstrated an overall response rate of 70.1% and a complete response rate of 33.3%. The median duration of response was 13.7 months, and the median progression-free survival was 9.1 months. Common adverse events included fatigue, maculopapular rash, and pyrexia. Notably, immune-related adverse events such as Guillain–Barré syndrome and polyradiculopathy were observed in a subset of patients. These findings suggest that Cami is a promising therapeutic option for patients with heavily pretreated cHL. Cami is now also being evaluated in other lymphoma subtypes [51].

Despite their success, antibody–drug conjugates (ADCs) face notable challenges, including antigen downregulation, heterogeneous expression, impaired internalization, and systemic toxicities such as neuropathy, cytopenias, and liver injury—particularly with MMAE-based ADCs. The beneficial bystander effect can also lead to off-target toxicity. To improve efficacy and safety, next-generation ADCs employ cleavable linkers sensitive to tumor-specific conditions (e.g., pH, redox potential) and alternative payloads like MMAF and PBD dimers, which offer potent cytotoxicity with reduced neurotoxicity. Advances in antibody engineering are enhancing internalization and antigen targeting [52,53,54].

Combination strategies with immune-checkpoint inhibitors, bispecific antibodies, and CAR-T-cell therapies are under active investigation. Preclinical data show that ADC-induced immunogenic cell death may enhance dendritic cell function and T-cell priming, boosting response to the PD-1/PD-L1 blockade. Clinically, ADCs are also being evaluated as bridging or salvage therapies around CAR-T-cell treatment, showing potential to address resistance due to immune dysfunction or antigen escape [55,56,57,58].

In conclusion, ADCs constitute a pivotal addition to the therapeutic arsenal for B-cell lymphomas. Their ability to deliver potent cytotoxic agents selectively to malignant cells has translated into durable remissions in patients with otherwise limited options. As advances in ADC technology continue, and as novel combination regimens are optimized, ADCs are poised to play an increasingly prominent role across the continuum of lymphoma therapy—from frontline regimens to salvage settings and maintenance strategies [59,60,61,62,63,64].

## 2. What Is Relapsed/Refractory Diffuse Large B-Cell Lymphoma?

DLBCL is the most prevalent and aggressive form of NHL, accounting for approximately 30–40% of all NHL cases worldwide. Its high incidence, combined with its rapid clinical progression, makes DLBCL a major public health concern. Although frontline chemoimmunotherapy—most commonly the R-CHOP regimen (rituximab, cyclophosphamide, doxorubicin, vincristine, and prednisone)—achieves remission in a substantial proportion of patients, a significant subset (approximately 30–40%) either relapses after initial treatment or fails to respond altogether.

The R/R setting is particularly difficult to manage due to the biological heterogeneity of the disease. Genetic alterations such as mutations or rearrangements involving *MYC*, *BCL2*, and *BCL6* contribute to aggressive tumor behavior and resistance to conventional therapies. In addition, immune escape mechanisms and changes in the tumor microenvironment further reduce treatment effectiveness.

Given DLBCL’s frequency and aggressiveness, there is an urgent need for innovative therapeutic strategies capable of overcoming resistance and improving patient outcomes. Emerging therapies—such as bispecific antibodies, antibody–drug conjugates, CAR-T-cell therapy, and small molecule inhibitors—represent a new era in the management of R/R DLBCL. These approaches offer the promise of more personalized, targeted treatment regimens that could improve survival and quality of life for a broader range of patients [65].

Recent therapeutic advances have improved outcomes in select patient populations. CAR-T-cell therapies—such as axicabtagene ciloleucel and tisagenlecleucel—have shown durable remissions in heavily pretreated cases. However, not all patients are eligible, and some relapse post-CAR-T. For these individuals, novel strategies like bispecific antibodies, allogeneic stem cell transplantation, and immune-checkpoint inhibitors (e.g., pembrolizumab) offer alternative avenues. Additionally, small molecules such as lenalidomide are under investigation for their immunomodulatory effects.

Prognosis in R/R DLBCL depends on multiple factors, including patient age, performance status, comorbidities, and molecular features. Therefore, personalized treatment approaches are essential to optimize outcomes and address the heterogeneous nature of the disease. Bispecific antibodies and antibody–drug conjugates in relapsed/refractory diffuse large B-cell lymphoma represent promising therapeutic strategies aimed at delivering more effective and individualized treatments.

Potential biomarkers that could predict a better response include tumor antigen expression levels, which directly influence BsAb and ADC binding efficacy and internalization. High target antigen density correlates with improved therapeutic response but may also increase on-target off-tumor toxicity. Additionally, tumor microenvironment characteristics such as immune-cell infiltration and checkpoint-molecule expression can modulate BsAb activity, particularly those engaging T cells, affecting both efficacy and immune-related adverse events. Pharmacogenomic factors, including polymorphisms in drug-metabolizing enzymes and Fc receptor genes, may alter ADC payload metabolism and antibody-dependent cellular cytotoxicity, respectively, impacting toxicity and efficacy profiles. Circulating biomarkers like soluble antigen fragments or circulating tumor DNA (ctDNA) levels could serve as non-invasive predictors of response and early indicators of resistance. Moreover, patient factors such as baseline organ function, prior treatment history, and immune status are critical for anticipating adverse events, especially in heavily pretreated populations. Integrating multi-omic data and real-time monitoring of these biomarkers in clinical trials may enable personalized dosing and patient selection, ultimately enhancing the therapeutic index of BsAbs and ADCs.

These innovative approaches hold the potential to significantly enhance both survival outcomes and patients’ quality of life, solving unmet clinical needs such as durable responses with manageable toxicity profiles and a pressing need for therapies that overcome resistance mechanisms to conventional chemoimmunotherapy and novel agents, as treatment-refractory disease often exhibits heterogeneous and complex resistance pathways [66,67].

### 2.1. Epcoritamab

Epcoritamab is a bispecific IgG1 antibody that simultaneously binds CD3 on T cells and CD20 on malignant B cells. By engaging these targets, it redirects T-cell-mediated cytotoxicity toward B-cell lymphomas, including aggressive subtypes such as DLBCL [68]. Epcoritamab has gained attention as a novel off-the-shelf immunotherapy option, especially for patients with R/R disease who have limited treatment options. The U.S. FDA granted accelerated approval in 2023 for adult patients with R/R DLBCL after at least two prior systemic therapies. This indication includes DLBCL transformed from indolent lymphomas such as follicular lymphoma. The EMA approved the therapy under conditional marketing authorization in the same year [69].

Epcoritamab is administered subcutaneously, a convenient alternative to intravenous infusions used with other bispecific antibodies or CAR-T-cell therapy. The subcutaneous route reduces administration complexity and is associated with a lower incidence of high-grade CRS. The suggested dosing protocol starts with a step-up approach during the first cycle, gradually increasing from 0.16 mg to 0.8 mg and then to 48 mg. This is followed by a maintenance dose of 48 mg once weekly for 12 weeks, then every two weeks up to week 48, and subsequently once a month until either disease progression or unacceptable toxicity occurs. To reduce the risk of CRS and ICANS, premedication with corticosteroids, antihistamines, and antipyretics is required during the initial doses.

Clinical evidence for epcoritamab efficacy stems from the pivotal Phase I/II EPCORE NHL-1 trial. Conducted in heavily pretreated patients with R/R large B-cell lymphoma, including DLBCL, the trial demonstrated an ORR of approximately 63%, with a CR rate of 38–39%. The median duration of response was approximately 15 months, with manageable safety concerns. Most CRS cases were Grade 1–2 and occurred early in treatment [70].

Several ongoing trials are investigating epcoritamab in broader clinical contexts. The Phase III EPCORE DLBCL-1 trial is comparing epcoritamab plus R-CHOP versus R-CHOP alone in previously untreated DLBCL. Additional trials are exploring epcoritamab in combination with agents like lenalidomide (NCT05660967), polatuzumab vedotin (NCT05283720), and chemotherapy backbones, aiming to optimize its integration into first-line and relapsed settings.

The Phase II, open-label, multicenter trial NCT05660967 is designed to investigate the efficacy and safety of epcoritamab, administered either as monotherapy or in combination with lenalidomide, in elderly patients with newly diagnosed CD20-positive DLBCL who are not candidates for anthracycline-based chemotherapy. The trial’s primary objectives include evaluating progression-free survival, overall response rate, and the safety profiles associated with each treatment arm. Epcoritamab’s bispecific engagement of CD3 and CD20 facilitates T cell-mediated cytotoxicity against malignant B cells, while lenalidomide’s immunomodulatory properties are anticipated to enhance this anti-tumor response. This study is particularly relevant for patients aged ≥75 years, or those ≥70 with substantial comorbidities, offering a potential frontline therapeutic option for a population with limited treatment alternatives.

NCT05283720 is a Phase II, open-label, multicenter study investigating the safety and efficacy of polatuzumab vedotin (Pola) in combination with bendamustine and rituximab (BR) for patients with relapsed or refractory DLBCL. In earlier trials, Pola-BR demonstrated a higher ORR and CR rate compared to BR alone.

These studies may further expand the role of epcoritamab across the treatment continuum in B-cell lymphomas.

### 2.2. Glofitamab

Glofitamab is a CD20 × CD3 bispecific monoclonal antibody that has emerged as a novel immunotherapy for R/R DLBCL. It engages both CD3 on T cells and CD20 on B cells, promoting T cell-mediated cytotoxicity against malignant B cells. It received accelerated approval from the FDA in June 2023 for the treatment of adult patients with R/R DLBCL or large B-cell lymphoma (LBCL) arising from indolent lymphoma after two or more lines of systemic therapy. The approval was based on the results of the pivotal NP30179 trial, which demonstrated significant efficacy in a heavily pretreated population [71].

Glofitamab is administered intravenously with a unique fixed-duration, step-up dosing regimen. Patients receive a pre-treatment dose of obinutuzumab (1000 mg IV on Day 7 of Cycle 1) to reduce tumor burden and mitigate the CRS risk, followed by step-up doses of glofitamab: 2.5 mg on Cycle 1 Day 1, 10 mg on Cycle 1 Day 8, and 30 mg on Day 1 of subsequent 21-day cycles, for a total of 12 cycles over approximately 9 months. This fixed course of therapy is distinct from many continuous-treatment regimens, offering a potentially more convenient and cost-effective treatment option [72].

The Phase I/II NP30179 study, which enrolled patients with R/R LBCL, demonstrated an ORR of approximately 50%, with a CR rate of 35%. Notably, responses were durable, with a median duration of response exceeding 18 months in those achieving CR. The safety profile was consistent with other T cell-engaging bispecifics, with CRS as the most common adverse event, mostly low-grade and manageable with supportive care and tocilizumab when necessary [73].

Several ongoing trials are investigating glofitamab in earlier lines of therapy and in combination regimens. For example, studies are evaluating its use with chemotherapy backbones such as CHOP or novel agents like polatuzumab vedotin (NCT06071871). These trials aim to determine whether glofitamab can enhance efficacy and potentially replace or supplement more intensive treatments like CAR-T-cell therapy in aggressive B-cell lymphomas such as DLBCL.

### 2.3. Polatuzumab Vedotin

Polatuzumab vedotin is an ADC that targets CD79b, a component of the B-cell receptor complex expressed on most B-cell malignancies, including DLBCL. The drug consists of a monoclonal antibody linked to MMAE, a cytotoxic microtubule inhibitor. Upon binding to CD79b, polatuzumab is internalized into the malignant B cell, releasing MMAE and inducing apoptosis. Developed by Genentech/Roche, polatuzumab vedotin has become an important treatment option in both relapsed/refractory and frontline settings for DLBCL [74].

Polatuzumab received accelerated FDA approval in 2019 in combination with bendamustine and rituximab (Pola–BR) for adult patients with relapsed or refractory DLBCL after at least two prior lines of therapy. This was based on data from the Phase Ib/II GO29365 trial, which demonstrated improved ORR and overall survival (OS) compared to BR alone. In 2023, polatuzumab vedotin was also approved for first-line treatment of newly diagnosed DLBCL in combination with rituximab, cyclophosphamide, doxorubicin, and prednisone (Pola-R-CHP), based on the pivotal Phase III POLARIX trial [75].

Polatuzumab vedotin is typically administered at a dose of 1.8 mg/kg intravenously every 21 days, commonly for six cycles when combined with the R-CHP regimen. In the Pola–BR protocol, the same dosing schedule is applied, also over a 21-day cycle for up to six cycles. Overall, treatment is generally well tolerated; however, frequently observed adverse events include peripheral neuropathy, neutropenia, and gastrointestinal disturbances, which are usually manageable through dose adjustments and appropriate supportive measures [76].

Ongoing clinical trials are exploring polatuzumab in a variety of combination regimens and treatment settings. Trials are evaluating its use with bispecific antibodies like glofitamab (NCT06071871) and epcoritamab (NCT05283720), as well as in CAR-T-cell bridging strategies and maintenance therapies. Research is also ongoing to determine whether polatuzumab-based regimens can be further optimized in high-risk DLBCL subtypes, including double-hit and primary refractory disease. These subgroups, characterized by aggressive biology and poor prognosis, often exhibit suboptimal responses to standard R-CHOP or salvage therapies. Incorporating polatuzumab vedotin, an anti-CD79b antibody–drug conjugate, into frontline or salvage regimens may enhance cytotoxic efficacy by directly delivering monomethyl auristatin E to malignant B cells. Studies have shown improved response rates and progression-free survival in combination with bendamustine and rituximab, and ongoing trials are evaluating its integration with intensive backbones, such as dose-adjusted EPOCH-R and novel agents like venetoclax or bispecific antibodies. Rational sequencing or combination with molecularly targeted agents may further optimize outcomes in DHL, particularly those harboring MYC and BCL2 and/or BCL6 rearrangements. Additionally, for primary refractory DLBCL, early introduction of polatuzumab may overcome chemoresistance and facilitate transition to curative modalities. Personalized, biomarker-guided strategies incorporating polatuzumab-based regimens hold promise to address the unmet needs in these high-risk populations, warranting continued investigation in prospective, subtype-specific clinical trials. These studies aim to enhance long-term outcomes and potentially reduce reliance on stem-cell transplantation and CAR-T-cell therapy.

### 2.4. Loncastuximab

Loncastuximab tesirine is an ADC that targets CD19, a surface antigen broadly expressed on B-cell malignancies, including DLBCL. Developed by ADC Therapeutics, loncastuximab is composed of a humanized monoclonal antibody conjugated to a PBD dimer toxin, which induces DNA crosslinking and cell death upon internalization into the tumor cell. This targeted mechanism allows selective delivery of a potent cytotoxic payload to malignant B cells while sparing healthy tissue [77].

In April 2021, loncastuximab tesirine received accelerated approval from the U.S. FDA for use in adult patients with relapsed or refractory diffuse large B-cell lymphoma (R/R DLBCL), including high-grade B-cell lymphoma, following at least two prior systemic treatment regimens. This regulatory decision was supported by data from the Phase II LOTIS-2 trial, a single-arm, open-label study involving heavily pretreated patients with aggressive B-cell malignancies. In the trial, loncastuximab achieved an overall response rate (ORR) of approximately 48%, with a complete response (CR) rate of 24%. Notably, clinical responses were observed across multiple high-risk patient subgroups. The median duration of response was approximately 10 months, highlighting the agent’s meaningful anti-tumor activity in a challenging treatment-refractory population.

The recommended dosing schedule involves intravenous administration at 0.15 mg/kg every 3 weeks for the first two cycles, followed by 0.075 mg/kg every 3 weeks for subsequent cycles until disease progression or unacceptable toxicity. Corticosteroid premedication is required to mitigate the risk of edema and capillary leak syndrome, which are known side effects. Other common adverse events include fatigue, elevated liver enzymes, neutropenia, and thrombocytopenia, though most toxicities are manageable with supportive care or dose modifications [78].

Several ongoing clinical trials aim to expand the role of loncastuximab in B-cell malignancies. LOTIS-3 is a Phase Ib/II study evaluating loncastuximab in combination with ibrutinib in R/R DLBCL, while LOTIS-5 is a randomized Phase III trial comparing loncastuximab plus rituximab versus standard immunochemotherapy in transplant-ineligible patients. In detail, the LOTIS-5 trial is a Phase III, randomized study evaluating loncastuximab tesirine combined with rituximab (Lonca-R) versus standard immunochemotherapy with rituximab, gemcitabine, and oxaliplatin (R-GemOx) in R/R DLBCL. Loncastuximab tesirine is an antibody–drug conjugate targeting CD19, delivering a cytotoxic payload selectively to malignant B cells. Part 1 of LOTIS-5 involved 20 patients receiving Lonca-R, demonstrating an overall response rate of 80% with a complete response rate of 50%. The median duration of response was 8.0 months, and progression-free survival was 8.3 months. Safety data revealed manageable adverse events, primarily neutropenia and elevated gamma-glutamyltransferase, with no new safety concerns. Part 2 is a larger randomized comparison enrolling approximately 330 patients, aiming to establish the efficacy and safety of Lonca-R versus R-GemOx. This trial explores a fixed-duration, chemo-free approach, potentially offering improved tolerability and quality of life for patients with R/R DLBCL. If successful, Lonca-R may represent a new standard of care, expanding treatment options beyond conventional chemotherapy for this high-risk population. Ongoing data from LOTIS-5 will clarify its role in clinical practice. These studies may establish loncastuximab as part of combination regimens and further validate its role across the DLBCL treatment spectrum.

Table 1 reports ongoing studies with epcoritamab, glofitamab, polatuzumab, and loncastuximab.

## 3. Future Perspectives

BsAbs and ADCs represent rapidly evolving therapeutic modalities in hematologic malignancies, with promising future perspectives. BsAbs like odronextamab, a CD20 × CD3 bispecific antibody, have demonstrated encouraging efficacy in relapsed or refractory B-cell non-Hodgkin lymphoma by redirecting T-cell cytotoxicity toward malignant B cells, yielding high overall and complete response rates. Advances in engineering have improved their safety profiles by reducing cytokine release syndrome and neurotoxicity, facilitating outpatient administration and earlier treatment lines.

Personalized treatment approaches incorporating biomarkers may guide patient selection, maximizing therapeutic benefit while minimizing toxicity. Overall, ongoing clinical trials and translational research are poised to expand the role of BsAbs and ADCs as integral components of precision medicine in relapsed/refractory NHL.

### 3.1. Odronextamab in DLBCL

Odronextamab is a promising bispecific antibody currently under investigation for the treatment of R/R DLBCL. The mechanism of action of odronextamab involves the simultaneous targeting of CD3 on T cells and CD20 on B cells, effectively redirecting T-cell cytotoxicity toward the malignant B cells. This bispecific approach aims to enhance immune-mediated tumor elimination while circumventing the need for traditional chemotherapy [64,79].

Preclinical in vivo studies of odronextamab have yielded valuable data regarding its anti-tumor efficacy and safety characteristics. The agent has been assessed in multiple xenograft mouse models engrafted with human B-cell malignancies. These investigations demonstrated potent T cell-mediated cytotoxicity leading to efficient tumor-cell elimination, alongside favorable pharmacokinetic and toxicity profiles. Dose-escalation analyses were conducted to define optimal therapeutic windows, with careful monitoring of cytokine release and immune activation parameters. Collectively, these in vivo findings support the clinical development of odronextamab, underscoring its ability to activate immune effector mechanisms and generate durable anti-tumor effects while maintaining an acceptable safety margin.

Therapeutic indications for odronextamab focus primarily on adult patients with DLBCL who have not responded to, or have relapsed after, previous lines of treatment, including chemotherapy and CAR-T-cell therapy. These patients face poor prognoses, and available therapies often offer limited survival benefits. By targeting both CD3 and CD20, odronextamab offers a novel approach that harnesses the immune system to fight the disease more effectively. Its mechanism is especially crucial for patients who have already received multiple lines of treatment without achieving durable remission [80].

The treatment schedule for odronextamab typically involves an initial phase of dose escalation to assess safety and tolerability, followed by a maintenance phase where the antibody is administered intravenously at a fixed dose. The exact dosing regimen is individualized based on the patient’s response and tolerability, but in clinical trials, it has generally been administered weekly for the first few cycles and then reduced to bi-weekly or monthly intervals.

Ongoing clinical trials are exploring the efficacy and safety of odronextamab in DLBCL, both as a monotherapy and in combination with other treatments. These trials include pivotal Phase I/II studies (NCT02995617) evaluating the antibody’s activity in relapsed/refractory DLBCL and comparing its performance against standard therapies. Early results have demonstrated promising response rates, with many patients achieving partial or complete remission. The ongoing studies are crucial in defining the optimal dosing schedule, long-term outcomes, and potential synergies with other therapeutic agents, such as immune-checkpoint inhibitors or chemotherapy regimens.

To summarize, odronextamab marks a notable advancement in the management of relapsed or refractory DLBCL, providing renewed hope for patients with limited treatment options [81].

### 3.2. Glofitamab in Mantle-Cell Lymphoma

Bispecific antibodies represent an emerging therapeutic modality also in the treatment of mantle-cell lymphoma (MCL), a rare and aggressive subtype of non-Hodgkin lymphoma characterized by poor long-term prognosis and frequent relapse after standard chemoimmunotherapy. Recent clinical trials have focused on the safety, efficacy, and tolerability of BsAbs targeting both malignant B cells and T cell-activating receptors, capitalizing on immune-mediated cytotoxicity to enhance anti-tumor responses. Among these, the ongoing Phase I/II studies NCT06054776, NCT07003295, and NCT06252675 are particularly noteworthy for their innovative approaches in targeting MCL.

The trial NCT06054776 evaluates the safety, tolerability, and efficacy of a combination regimen of acalabrutinib, obinutuzumab, and glofitamab in patients with relapsed or refractory MCL. Acalabrutinib is a Bruton tyrosine kinase (BTK) inhibitor that disrupts B-cell receptor signaling, inhibiting lymphoma cell growth. Obinutuzumab is an anti-CD20 monoclonal antibody that enhances immune-mediated cytotoxicity. Glofitamab is a bispecific antibody targeting CD20 on B cells and CD3 on T cells, promoting T-cell activation and direct tumor-cell lysis. The primary objectives include assessing the safety profile of acalabrutinib plus glofitamab (safety lead-in) and estimating the CR rate in Phase II. Secondary objectives involve evaluating MRD negativity, time to response, duration of response (DoR), progression-free survival (PFS), OS, patient-reported quality of life, and treatment-related toxicities. Patients receive acalabrutinib orally twice daily, obinutuzumab IV during Cycle 1, and glofitamab IV on specified days of each 21-day cycle for up to 12 cycles. Post-treatment follow-up includes imaging, biopsies, and laboratory monitoring, continuing up to 4 years.

The NCT07003295 Phase II clinical trial investigates the safety, tolerability, and efficacy of glofitamab and obinutuzumab in patients with relapsed or refractory MCL following prior CD19-directed CAR-T-cell therapy. Glofitamab is a bispecific antibody targeting both CD3 on T cells and CD20 on B cells, facilitating T cell-mediated cytotoxicity against malignant B cells. Obinutuzumab, a monoclonal anti-CD20 antibody, may enhance immune-mediated clearance of lymphoma cells and reduce immune-related toxicity. The primary objective is to assess the ORR to glofitamab in this post-CAR-T-cell setting. Secondary objectives include evaluating CR rates, PFS, OS at 24 months, and the incidence of grade 3–4 CRS and neurologic toxicity. Additionally, pharmacokinetic correlations between glofitamab clearance and treatment response are explored. Patients receive IV obinutuzumab on day 1 (±day 2) of Cycle 1, followed by glofitamab on Days 8 and 15 of Cycle 1 and on Day 1 of Cycles 2–12. Treatment cycles repeat every 21 days. Follow-up continues every 3 months for up to 2 years.

Lastly, the NCT06252675 Phase II clinical trial evaluates the safety, tolerability, and preliminary efficacy of the combination of glofitamab and pirtobrutinib in patients with R/R MCL. Glofitamab and obinutuzumab are monoclonal antibodies; obinutuzumab is used pre-treatment to mitigate immune-related adverse events. Pirtobrutinib is a non-covalent BTK inhibitor that blocks signaling pathways critical for MCL cell proliferation. The primary objectives are to assess the safety profile of the combination in the first six patients and to determine the CR rate in the overall population. Secondary endpoints include PFS, OS, ORR, DoR, CR without measurable residual disease (CRMRD-) rate, time to CRMRD-, and treatment-free interval post-CRMRD-. Exploratory analyses aim to correlate baseline tumor genetic and immune profiles with treatment outcomes and to evaluate pharmacodynamic resistance markers. Quality-of-life metrics and additional time-to-CRMRD- analyses are also included. Patients receive obinutuzumab IV (Cycle 1), followed by glofitamab IV and daily oral pirtobrutinib over 21-day cycles for up to 12 cycles. Imaging, biopsy, and blood analyses are conducted periodically. Post-treatment follow-up occurs every 3 months for 2 years.

Together, these trials underscore the growing interest in BsAbs as a promising class of immunotherapies for MCL. They highlight the need for optimized dosing strategies, biomarker development, and combination regimens to maximize therapeutic efficacy while minimizing immune-related toxicities in this challenging disease context. Table 2 reports ongoing studies with odronextamab and glofitamab.

## 4. Conclusions

Bispecific antibodies represent a significant advancement in cancer immunotherapy by targeting two distinct antigens simultaneously, thereby enhancing tumor specificity. Although initially developed in the 1960s, early clinical application was hindered by issues related to molecular stability and immunogenicity. Advances in recombinant DNA technology have improved the pharmacokinetic and safety profiles of BsAbs, enabling their clinical viability.

The approval of blinatumomab in 2014 for B-precursor acute lymphoblastic leukemia (ALL) validated the therapeutic potential of BsAbs and catalyzed the development of agents such as glofitamab, epcoritamab, and mosunetuzumab for relapsed/refractory B-cell non-Hodgkin lymphoma. However, clinical challenges such as CRS, ICANS, and antigen downregulation remain significant. Combination approaches involving checkpoint inhibitors and tumor microenvironment modulation are under investigation to overcome these limitations. Epcoritamab offers a favorable safety profile and subcutaneous administration, making it suitable for outpatient management, while glofitamab may be preferred in settings favoring fixed-duration therapy.

Antibody–drug conjugates like polatuzumab vedotin and loncastuximab tesirine have also demonstrated efficacy in relapsed/refractory DLBCL, particularly in patients unresponsive to conventional regimens. BsAbs and ADCs are being actively studied in pre- and post-CAR-T-cell therapy settings, with treatment sequencing increasingly tailored to disease biology and patient characteristics.

The future of B-cell lymphoma therapy lies in personalized, biomarker-driven approaches that optimize efficacy while minimizing toxicity and addressing resistance mechanisms.

## Figures and Tables

**Table 1:** Ongoing clinical trials with epcoritamab, glofitamab, polatuzumab, and loncastuximab.

Trial Name/NCT Number	Phase	Therapeutic Combination	Indication	Objective
NCT06492837	Phase I	Mosunetuzumab + Zanubrutinib	B-NHL	Assess early safety/efficacy data
NCT05410418	Phase II	Mosunetuzumab + Polatuzumab Vedotin	B-NHL	Expand indications for combination therapies
NCT06453044	Phase I	Mosunetuzumab + Polatuzumab Vedotin	B-NHL	Evaluate efficacy in early-line or refractory cases
EPCORE NHL-1	Phase I/II	Epcoritamab monotherapy	R/R LBCL	Evaluate efficacy/safety in heavily pretreated patients
EPCORE DLBCL-1	Phase III	Epcoritamab + R-CHOP vs. R-CHOP	Previously untreated DLBCL	Compare efficacy with standard chemoimmunotherapy
NCT05660967	Phase II	Epcoritamab + Lenalidomide	B-NHL	Assess combination in relapsed/refractory settings
NCT05283720	Phase I/II	Epcoritamab + Polatuzumab Vedotin	B-NHL	Explore safety/efficacy in combination regimens
NCT06071871	Phase I/II	Glofitamab + Polatuzumab Vedotin ± Chemotherapy	Aggressive B-NHL	Assess efficacy in earlier lines or in combination regimens
LOTIS-3	Phase Ib/II	Loncastuximab + Ibrutinib	R/R DLBCL	Evaluate combination therapy efficacy and safety
LOTIS-5	Phase III	Loncastuximab + Rituximab vs. standard immunochemotherapy	Transplant-ineligible R/R DLBCL	Compare combination therapy to standard immunochemotherapy

**Table 2:** Ongoing clinical trials with odronextamab and glofitamab.

Trial Name/NCT Number	Phase	Therapeutic Combination	Indication	Objective
NCT02995617	Phase I/II	Odronextamab monotherapy	R/R DLBCL	Evaluate efficacy in pretreated patients
NCT06054776	Phase I/II	Glofitamab + acalabrutinib+ Obinutuzumab	R/R MCL	Evaluate safety and MRD negativity, DoR, PFS, and OS in pretreated patients
NCT07003295	Phase I/II	Glofitamab + obinutuzumab	R/R MCL	Evaluate ORR, CR rates, PFS, OS at 24 months, and the incidence of grade 3–4 CRS in post-CAR-T patients
NCT06252675	Phase II	Glofitamab + pirtobrutinib	R/R MCL	Evaluate efficacy/safety and tolerability in pretreated patients

## Abbreviations

ADCs, antibody–drug conjugates; ADCC, antibody-dependent cellular cytotoxicity; ALL, acute lymphoblastic leukemia; AML, acute myeloid leukemia; BiTEs, bispecific T-cell engagers; BsAbs, bispecific antibodies; BTK, Bruton’s tyrosine kinase; CAR-T, chimeric antigen receptor T-cell; cHL, classical Hodgkin lymphoma; CR, complete response; CRMRD-, CR without measurable residual disease; CRS, cytokine release syndrome; CTLs, cytotoxic T lymphocytes; DARTs, dual-affinity re-targeting; DLBCL, diffuse large B-cell lymphoma; DoR, duration of response; EMA, European Medicines Agency; FDA, Food and Drug Administration; GO, gemtuzumab ozogamicin; ICANS, immune effector cell-associated neurotoxicity syndrome; LBCL, large B-cell lymphoma; MCL, mantle-cell lymphoma; MHC, major histocompatibility complex; MMAE, monomethyl auristatin E; MMAF, monomethyl auristatin F; MRD, minimal residual disease; NHL, non-Hodgkin lymphoma; ORR, overall response rate; OS, overall survival; PBD, pyrrolobenzodiazepine; PFS, progression-free survival; PMBCL, primary mediastinal large B-cell lymphoma; POD-24, progression of disease within 24 months; R/R, relapsed/refractory; sALCL, systemic anaplastic large-cell lymphoma; TAMs, tumor-associated macrophages; TandAbs, tandem diabodies; TCR, T-cell receptor; TME, tumor microenvironment; Tregs, regulatory T cells.
